# Impact of a passive social marketing intervention in community pharmacies on oral contraceptive and condom sales: a quasi-experimental study

**DOI:** 10.1186/s12889-015-1495-x

**Published:** 2015-02-13

**Authors:** Karen B Farris, Mary L Aquilino, Peter Batra, Vince Marshall, Mary E Losch

**Affiliations:** University of Michigan College of Pharmacy, Ann Arbor, MI USA; University of Iowa College of Public Health, Iowa City, IA USA; University of Northern Iowa Center for Social & Behavioral Research, Cedar Falls, IA USA

**Keywords:** Unintended pregnancy, Community pharmacy, Condoms, Oral contraceptives

## Abstract

**Background:**

Almost 50% of pregnancies in the United States are unwanted or mistimed. Notably, just over one-half of unintended pregnancies occurred when birth control was being used, suggesting inappropriate or poor use or contraceptive failure. About two-thirds of all women who are of reproductive age use contraceptives, and oral hormonal contraceptives remain the most common contraceptive method. Often, contraceptive products are obtained in community pharmacies. The purpose of this study was to determine whether a pharmacy-based intervention would impact sales of contraceptive products in pharmacies.

**Methods:**

This study was conducted in Iowa and used a quasi-experimental design including 55 community pharmacies (independent and grocery) in 12 counties as the intervention and 32 grocery pharmacies in 10 counties as a comparison group. The passive intervention was focused towards 18–30 year old women who visited community pharmacies and prompted those of childbearing age to “plan your pregnancy” and “consider using birth control”. The intervention was delivered via educational tri-fold brochures, posters and ‘shelf talkers.’ Data sources for evaluation were contraceptive sales from intervention and comparison pharmacies, and a mixed negative binomial regression was used with study group*time interactions to examine the impact of the intervention on oral contraceptive and condom sales. Data from 2009 were considered baseline sales.

**Results:**

From 2009 to 2011, condom sales decreased over time and oral contraceptives sales showed no change. Overall, the units sold were significantly higher in grocery pharmacies than in independent pharmacies for both contraceptive types. In the negative binomial regression for condoms, there was an overall significant interaction between the study group and time variables (p = 0.003), indicating an effect of the intervention, and there was a significant slowing in the drop of sales at time 3 in comparison with time 1 (p < 0.001). There was a statistically significant association between pharmacy type and study group, where the independent intervention pharmacies had a higher proportion of stores with increases in condom sales compared to grocery pharmacies in the intervention or comparison group.

**Conclusions:**

A passive community pharmacy-based public health intervention appeared to reduce the decrease in condom sales from baseline, particularly in independent pharmacies, but it did not impact oral contraceptive sales.

## Background

Almost 50% of pregnancies in the United States are unwanted or mistimed [1]. Women who are poor, age 18–24, cohabiting and minority are more likely to experience unintended pregnancies [1]. Unintended pregnancy is associated with delayed prenatal care and poor outcomes [2-4]. Evidence suggests that reducing risky behavior, promoting use of effective contraception and improving appropriate use of all contraceptive methods remain important public health goals. Notably, just over one-half of unintended pregnancies occurred when birth control was being used [5].

In the United States, about two-thirds of all women who are of reproductive age use contraceptives, and oral contraceptives remain the most common hormonal method of contraception [2]. In 2010, oral contraceptives and female sterilization were the two most common contraceptive methods among women who practiced contraception in the U.S., and this has been true since 1982 [6,7]. Among all U.S. women 15–44 years old who practice contraception, 17% use oral contraceptives, 3.5% use an intrauterine device, 2.4% use a hormonal injectable, 1.3% use a vaginal ring and < 0.5% use either patch or implant [7].

Often, prescription or nonprescription contraceptive products, primarily hormonal methods, are obtained in community pharmacies. In the United States, oral contraceptives, contraceptive patches, vaginal rings and contraceptive injections are available via prescription in pharmacies, yet oral contraceptives are the most widely used product. Condoms and spermicides are available over-the-counter, and condoms are widely available in other types of retail outlets. At the time of this study, emergency contraception was available in pharmacies from behind the counter, requiring pharmacists to counsel about its use.

Farris et al. outlined roles of pharmacists in reducing unintended pregnancy, yet pharmacists typically dispense and sell contraceptive products with little additional interaction with consumers/patients [8]. In terms of emergency contraception, access has been an issue with some pharmacists refusing to sell it, although attitudes seemed to have improved since the early 2000s [8,9]. Over the past two decades, laws in states have taken various strategies to address access to contraceptives from requiring pharmacies to fill all valid prescriptions to expressly allowing pharmacists to refuse to dispense emergency contraception [10]. Admittedly, contraception in the United States is considered by some to be an ethical issue, and some pharmacists do refuse to dispense contraceptives and sell emergency contraceptives [10].

The vast majority of pharmacists and pharmacies may be a valuable non-traditional venue for disseminating information about improving effective contraceptive use and reducing unintended pregnancy [8,9,11-15]. In fact, pharmacists are considered an accessible healthcare provider because they are well-trained, available in rural areas and do not typically require appointments. Pharmacies and pharmacists have not capitalized upon this potential role, although patient-oriented practices are discussed widely in the pharmacy literature [14-18].

We are aware of no other study using a social marketing approach in community pharmacies to impact contraceptive sales [8,15]. Previous studies in pharmacy have primarily focused on access to emergency contraception. In addition, progesterone injections have been administered in pharmacies. Finally, new models of contraceptive delivery have been tested, whereby pharmacists working under collaborative practice agreements are able to prescribe hormonal contraceptives [19,20]. In fact, the American College of Clinical Pharmacy Women’s Health Practice and Research Network advocates changing the status of oral contraceptives from prescription to over-the-counter status in licensed pharmacies while a pharmacist is on duty [15].

Because of their potential public health role, community pharmacies were one component of the Iowa Initiative to Reduce Unintended Pregnancies active in Iowa from 2007–2012. The overall program sought to use policy, access to healthcare services including long-acting reversible contraceptives and research interventions to impact the rate of unintended pregnancy in the state [21]. There were five research interventions examined in the program, all focused to persuade adult women 18–30 years old to seek and purchase contraceptives, particularly long-acting reversible contraceptives, if they wished to delay or prevent pregnancy. Across Iowa, there were about 183,000 women age 15–29 requiring contraceptives [22]. One of the five interventions was in the community pharmacy, a non-traditional avenue for public health initiatives. The purpose of this analysis was to determine whether a pharmacy-based intervention would impact sales of contraceptive products in pharmacies.

## Methods

### Design/setting

We used a quasi-experimental observational design to evaluate the intervention in Iowa pharmacies. This study was approved by the Institutional Review Boards at the University of Northern Iowa, University of Iowa and University of Michigan. Sixty pharmacies began the intervention that ran from Fall 2009 to Fall 2011. We fully implemented the intervention in 55 community pharmacies in 12 counties, and the pharmacies were either independently owned or located inside a mid-western grocery chain. We were able to recruit in specified counties established by the larger Iowa Initiative project, and we included those counties where we were able to recruit at least 25% of pharmacies. The pharmacies were recruited using an introductory letter, telephone contact and visit by study personnel, typically one of the co-principal investigators. Early in 2010, two pharmacies withdrew citing the amount of time to participate and three withdrew due to a customer complaint regarding the subject matter. We identified 10 counties in Iowa with similar demographics to counties where the intervention pharmacies resided. We identified 32 pharmacies in the same grocery chain in those 10 counties, and these pharmacies served as a comparison group. These control counties provided a means to monitor the impact of the other Iowa Initiatives on pharmacy contraceptive sales and to easily gather sales data.

Based on our initial survey of Iowa pharmacies, pharmacies in Iowa dispensed an average of 201 prescriptions per day but the range was broad (standard deviation was 100). On average they employed 1.67 (s.d. 0.77) pharmacists and 2.44 (s.d. 1.47) pharmacy technicians. Independent pharmacies compared to grocery pharmacies tended to be more likely to have a private area to talk with patients, be less likely to stock male condoms, be more likely to place male condoms on shelf (not behind locked glass) and be less likely to dispense emergency contraception [11]. There are 99 counties in Iowa and approximately 700 community pharmacies in the state.

### Intervention

The intervention was comprised of seven sets of social-marketing materials placed in the intervention pharmacies at quarterly intervals, and pharmacy staff was aware of the intervention. The social marketing campaign in the pharmacies prompted women of childbearing age to “plan your pregnancy” and “consider using birth control”. The goal of the intervention in the pharmacy was to increase the uptake of contraceptive products, and we measured this potential effect in pharmacies by way of contraceptive sales. Information was delivered passively via educational tri-fold brochures, posters and ‘shelf talkers.’ Each quarter a free product such as lip balm, pen or chip clip with the study logo was set near the cash register so that any individual could chose to take it. The free products, posters and brochures were branded in a similar manner with the study logo. Materials were used passively and were not directly distributed to individuals purchasing contraceptive products. We chose to use a passive approach because there was no reimbursement available to sustain pharmacists’ expanded services. Anecdotally, pharmacists told us that they used the tri-fold brochure explaining hormonal contraceptives in their patient counseling.

**Figure 1:**
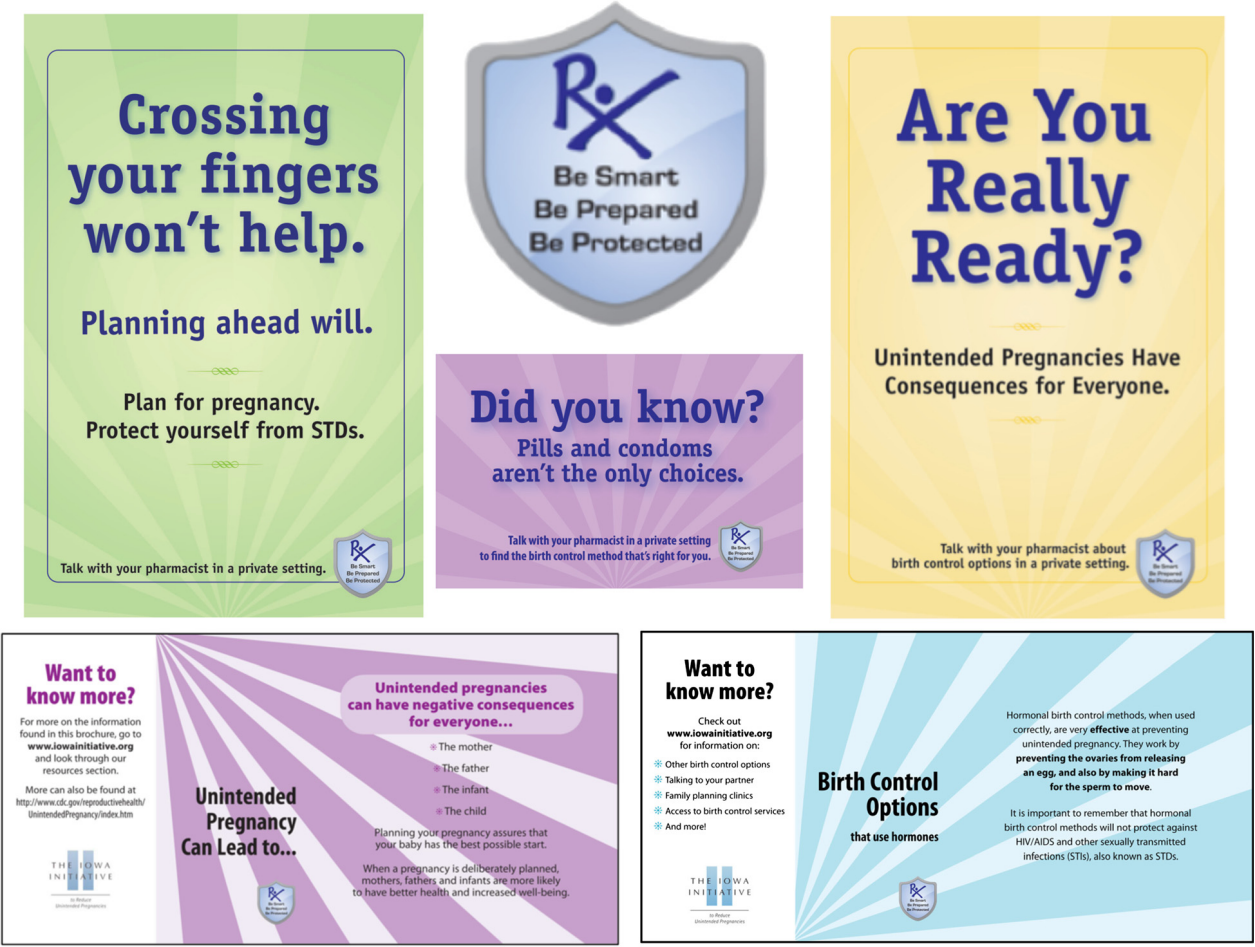
**Selected materials from the pharmacy-based social marketing campaign.**

The original social-marketing messages were based upon a previous campaign called Pharmacy Access Partnership [23]. For our project, selected materials were reviewed by seven Iowa community pharmacies in personal interviews, four focus groups of young adults and an Advisory Board (for the study) comprised of four community pharmacists and four young adult female consumers. The study team reviewed the original marketing materials and developed new messages, and the Advisory Board reviewed the final materials. In total, we produced 6 educational brochures, 7 shelf-talkers and 8 posters (Table 1 and Figure 1). Examples of messages included “Crossing your Fingers Won’t Help”, “50% of Pregnancies in Iowa are Not Planned” and “Did You Know? There Are More Choices than Pills or Condoms”. Each quarter the materials were different colors, but all materials during a quarter used the same color.

**Table 1:** **Social-marketing materials by quarter**

**Quarter**	**Informational brochures (titles)**	**Shelf talkers (message content)**	**Posters (message content)**	**Give-away**
1 and 5	Birth Control Options That Use Hormones	Crossing Your Fingers Won’t Help	Be Smart, Be Prepared, Be Protected	Lip balm, Pens
	Birth Control Options Without Hormones	Got Plans? Protect Yourself From STDs	Did You Know? 50% of Pregnancies In Iowa Are Not Planned	
		Did You Know? A Pharmacist Is Here To Talk		
2 and 6	Condoms	Did You Know? STDs Can Last A Lifetime	Did You Know? There Are More Choices Than Pills or Condoms	Hand Sanitizer, Emery Boards
	Talk With Your Partner About Contraceptives	50% of Pregnancies In Iowa Are Not Planned	Crossing Your Fingers Won’t Help	
3 and 7	Unintended Pregnancy Can Lead To…	Did You Know? There Are More Choices Than Pills or Condoms	Did You Know? A Pharmacist Is Here To Talk	Magnetic Clips, Post-It Notes
	Ask Your Pharmacist About Contraceptives	Be Smart, Be Prepared, Be Protected	Plan Ahead. Hope Won’t Help.	
4	Condoms	50% of Pregnancies In Iowa Are Not Planned	Did You Know? You Can Ask Your Pharmacist About Contraceptives.	Hand Sanitizer Spray
	Talk With Your Partner About Contraceptives	Crossing Your Fingers Won’t Help	Are You Really Ready?

Research assistants visited the pharmacies quarterly and changed the materials. Posters were placed on glass fronts or walls near the pharmacy department, shelf-talkers were placed near non-prescription contraceptives and educational brochures were placed near contraceptives, near other brochures or at the pharmacy cash register. Free products were also placed near the cash register. Research assistants maintained monthly contact with the pharmacy staff to ensure sufficient materials were available. Our research assistants documented that all free products with the study logo were distributed, over 600 brochures were distributed and each intervention pharmacy had posters and/or shelf talkers posted in their pharmacies each period of the intervention.

### Data sources

**Figure 2:**
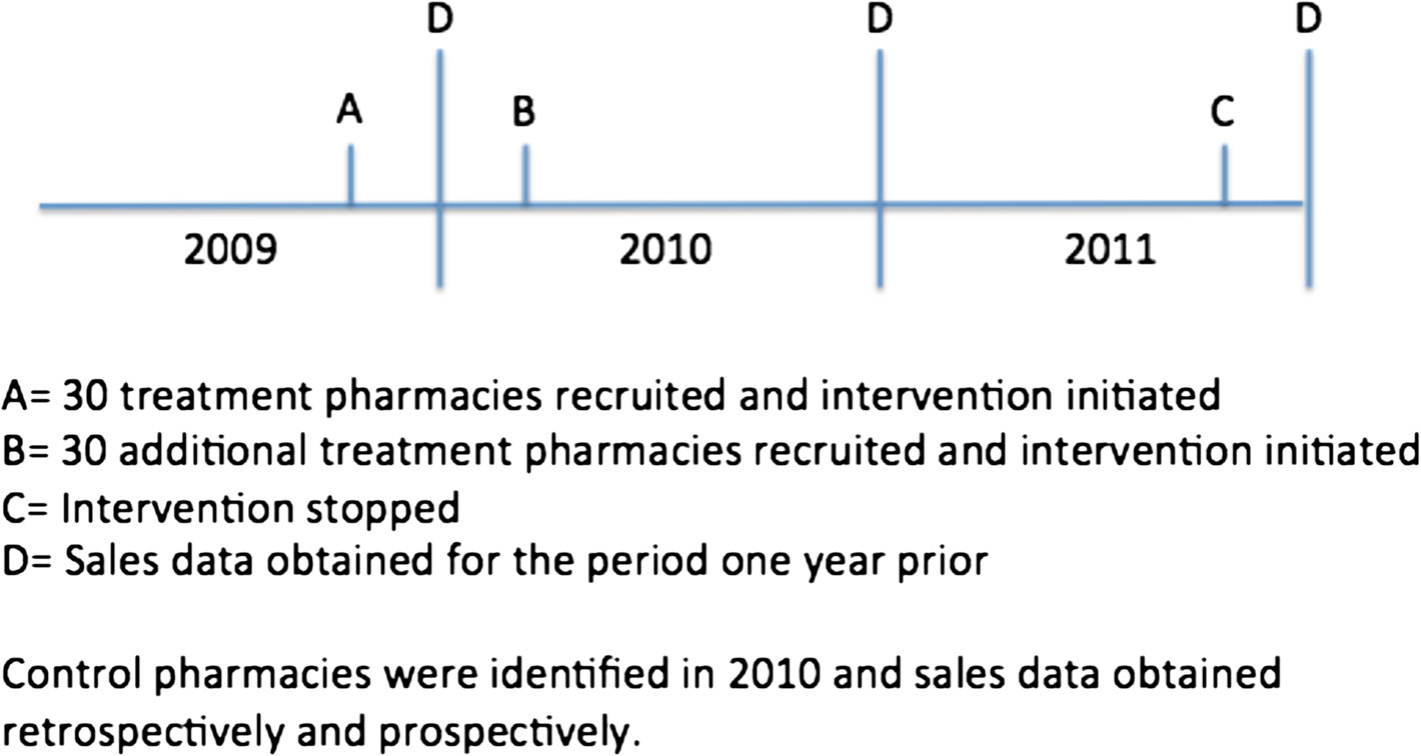
**Timeline of recruitment, intervention and data collection.**

The evaluation presented here uses contraceptive sales data from the intervention and comparison pharmacies. We gathered attitudinal and experience data from a random sample of young Iowa women as well as from a convenience sample in the intervention pharmacies, but the sales data allow us to control for the other Iowa Initiatives activities via the control group, and the sales data are an objective assessment of the intervention. We collected data from pharmacies of the sales of all hormonal contraceptives, but very few sales existed for products other than oral contraceptive and condoms. Long-acting reversible contraceptives are typically obtained from physician offices or clinics not pharmacies. The three time periods of contraceptive sales data corresponded to 2009, 2010, and 2011, and 2009 is the baseline. Using 2009 sales data as the baseline biases our results towards no effect, given that contraceptive sales could have been affected by the intervention in the last quarter of 2009. The grocery store data were collected from November 1 of the year prior to the stated year until October 31 of the stated year, and these dates represent less bias. The independent pharmacies had two different time periods, either the same as the grocery store or from January 1 to December 31 of the stated year, and these times varied because of different recruitment dates into the study (Figure 2).

The contraceptive sales data were count data designating the number of units sold in each pharmacy for each type of contraceptive. Several types of contraceptive products were obtained including condoms, oral contraceptives, emergency contraceptives, contraceptive patches, vaginal rings, contraceptive injections, female condoms, and spermicides. In the final analysis, condoms and oral contraceptives were considered because other sales were too sparse. The independent pharmacies sent yearly sales from forms created for hand-entry, and these data were entered into a spreadsheet and verified for accuracy. The contraceptive sales data from the grocery pharmacies were obtained from a central location. For analysis, we also recorded the county in which the store was located and whether the pharmacy was grocery or independent. Of the original 93 stores, 6 did not contribute data in the first two time periods, and 9 did not have data in the third time period, so the final analysis sample size was n = 84 (Table 2).

**Table 2:** **Contraceptive sales for intervention and comparison pharmacies** ^†^

	**Condoms (mean/standard deviation)**		**Oral contraceptives (mean/standard deviation)**	
	**Intervention**	**Comparison**	**Intervention**	**Comparison**
**All Pharmacies**				
2009	639(997)	1584(1698)	1107(922)	2014(1116)
2010	656(1044)	1282(1390)	1034(816)	1881(1045)
2011	376(476)	666(550)	1120(946)	1984(1046)
**Independent Pharmacies Only**				
2009	186 (516)	0	742(615)	344(NA)
2010	214 (792)	0	686(429)	441(NA)
2011	144 (272)	0	805(801)	461(NA)
**Grocery Pharmacies Only**				
2009	1426(1146)	1635(1700)	1741(1032)	2068(1091)
2010	1423(998)	1324(1393)	1640(974)	1928(1028)
2011	778(491)	687(545)	1667(947)	2033(1025)

^†^N analyzed for times 1 and 2 and 3 = 84 (intervention = 52 comparison = 32).

### Analysis

For descriptive analysis, we used mean and standard deviation. To examine whether there was any change in the intervention pharmacies compared to the comparison pharmacies, we used mixed negative binomial regressions for our primary analysis predicting condom and oral contraceptive sales (separately) blocked at the county level. The primary test of interest was the interaction between the study group (intervention versus comparison) and the time variable, which were both fixed effects. The type of pharmacy was adjusted with a random clustering effect, and the pharmacies were nested within pharmacy type (grocery or independent. In addition to testing for the interaction, we assessed the main effects for the models. We used (mean) interaction plots for visualization of the main effects and interactions. We used 2010 Census data to create an adjusted model, which considered median age, percent of females in the population, percent college educated and the mean persons per household for each county. Importantly, we conducted a separate analysis using grocery pharmacy data only, and used the same analytic approach. Our review of the sales data in grocery pharmacies indicated a significant drop-off in reported sales, i.e., no sales, in nine pharmacies, and we completed our analysis with and without those pharmacies to test their presence on any effects. Finally, we examined the number of pharmacies with negative or positive changes in sales over time. All analyses were done using SAS or R statistical software.

## Results

Comparing data from the time 1 baseline to times 2 and 3 during the study, overall condom sales decreased over time and oral contraceptives sales showed no change (Table 2). At all time periods, the units sold were significantly higher in the grocery pharmacies than in the independent pharmacies for both contraceptive types (two sample *t*-test p_condoms_ < 0.001, p_OC_ < 0.001). Over the three-year study, the average number of condoms sold per grocery pharmacy was 1213 (s.d. 1214) versus 176 (s.d. 555) for independent pharmacies. For oral contraceptives, the means per pharmacy were 1885 (s.d. 1022) versus 735 (s.d. 622) for 28-day units, respectively.

**Figure 3:**
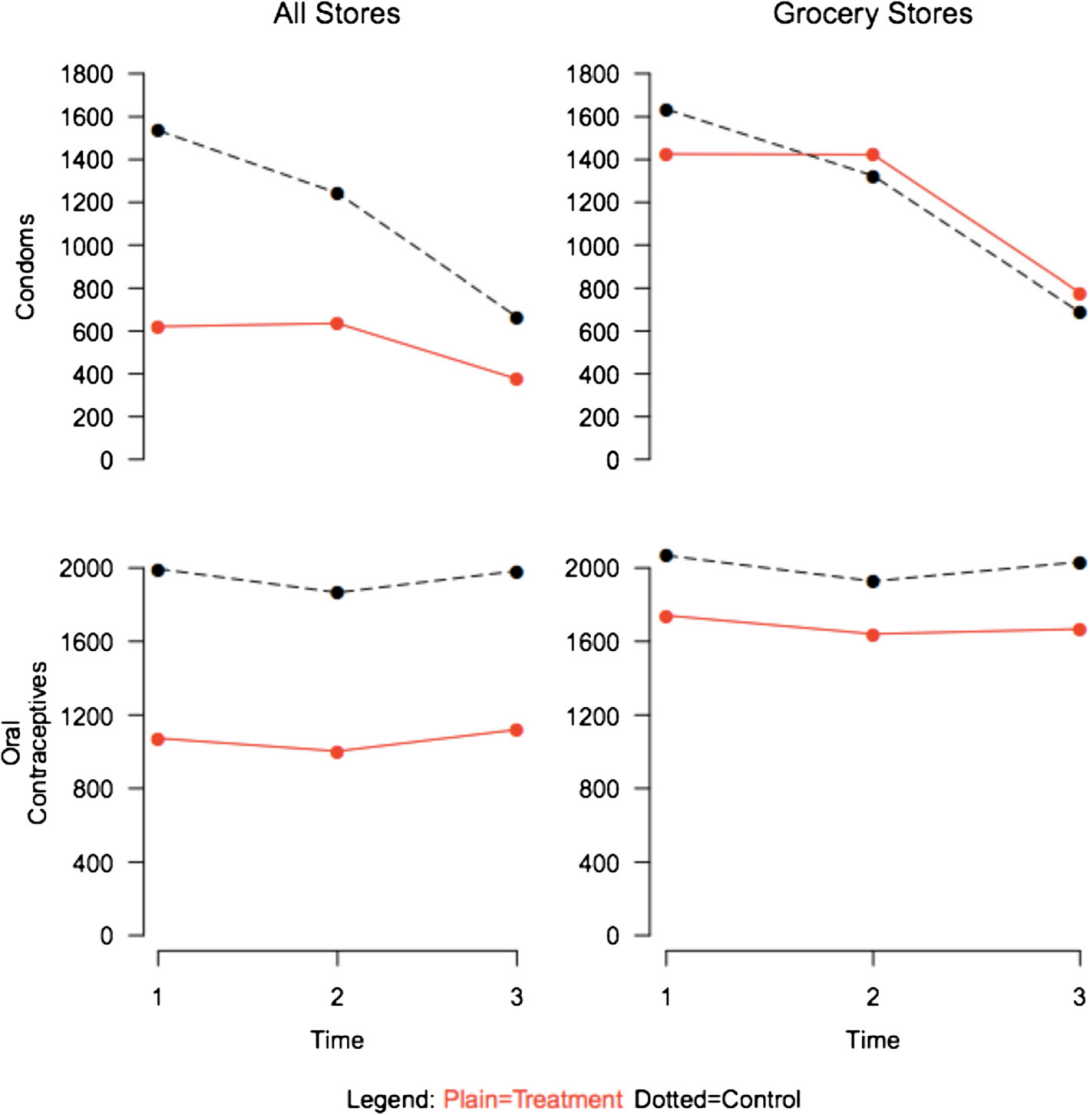
**Contraceptive sales interaction plots (Mean).**

The negative binomial regression for condoms was used to examine the impact of the intervention on condom sales by examining the interaction of study group by time. The interaction between the study group and time was statistically significantly different that zero (p = 0.003), indicating an effect of the intervention. The main effects showed that condom sales were lower in intervention pharmacies than in comparison pharmacies and decreased over time (Table 3). While condom sales dropped in both study groups, the rate of decline in the intervention pharmacies was less steep than in the comparison pharmacies. Specifically, at time 2, the difference in mean sales between intervention and comparison pharmacies was not significant (p = 0.283). There was, however significant slowing or decreased slope at time 3 versus time 1 (p < 0.001). This finding is also reflected in the pairwise interaction between times 2 and 3 (p = 0.020, Figure 3, top graphs). Similar results were seen for the study group by time interaction in the linear mixed models (results not shown). When we excluded the nine pharmacies with no sales toward the end of the reporting period from the regression, the overall interaction for study group by time remained statistically significant (p = 0.046), as did the interaction between times 1 and 3 (p = 0.003) (results not shown).

**Table 3:** **Contraceptive sales negative binomial regression results by study group and time**

	**Estimate** ^**†**^	**SE**	**LB**	**UB**	**t**	**P value**
**Condoms**						
intervention (vs. comparison)	−1.43	0.73	−2.87	0.01	−1.96	0.051
time 2 (vs. 1)	−0.05	0.07	−0.20	0.09	−0.74	0.461
time 3 (vs. 1)	**−0.46**	**0.07**	**−0.61**	**−0.32**	**−6.42**	**<.001**
time 3 (vs. 2)	**−0.41**	**0.07**	**−0.55**	**−0.27**	**−5.69**	**<.001**
intervention * time 2 (vs. 1)	0.16	0.14	−0.13	0.44	1.08	0.283
intervention * time 3 (vs. 1)	**0.50**	**0.14**	**0.21**	**0.78**	**3.42**	**<.001**
intervention * time 3 (vs. 2)	**0.34**	**0.14**	**0.06**	**0.62**	**2.35**	**0.020**
**Oral Contraceptives**						
intervention (vs. comparison)	**−0.74**	**0.22**	**−1.17**	**−0.32**	**−3.44**	**<.001**
time 2 (vs. 1)	−0.04	0.04	−0.11	0.04	−0.91	0.363
time 3 (vs. 1)	0.01	0.04	−0.07	0.09	0.02	0.842
time 3 (vs. 2)	0.04	0.04	−0.03	0.12	1.11	0.268
intervention * time 2 (vs. 1)	0.06	0.08	−0.10	0.21	0.73	0.465
intervention * time 3 (vs. 1)	0.03	0.08	−0.12	0.19	0.40	0.693
intervention * time 3 (vs. 2)	−0.03	0.08	−0.18	0.13	−0.34	0.737

^†^Main effect and interaction estimates and p values are computed with contrast tests in the regression.

Interaction terms reference category is always control.

LB = Lower Bound, UB = Upper Bound for a 95% Confidence Interval.

For oral contraceptives, there was no statistically significant overall interaction between study group and time (p = 0.765, indicating no effect of the intervention on oral contraceptive sales. The pairwise interactions were also not significant (Table 3, Figure 3). The study group by time interaction in the linear mixed models for oral contraceptives showed similar results to the negative binomial model (results not shown). In the adjusted models including four covariates from Census data, the model interaction results for study group by time were similar to our previous findings (p_condoms_ = 0.003, p_oral contraceptives_ = 0.765).

We repeated the same analysis using only grocery pharmacies in the intervention and comparison groups. In this analysis, there was no statistically significant interaction between the study group by time variables for any of the types of contraceptives (Table 4). To gain another view of the effect of the intervention on condom sales, we examined the number of pharmacies with decreases in condom sales compared to those with increases or no change (Table 5). There was a statistically significant association between pharmacy type and study group, and the independent intervention pharmacies appeared to have a higher proportion of stores with increases in condom sales compared to grocery pharmacies in the intervention or comparison group.

**Table 4:** **Grocery pharmacy contraceptive sales negative binomial regression results by study group and time**

	**Estimate** ^**†**^	**SE**	**LB**	**UB**	**t**	**P value**
**Condom Sales**						
intervention (vs. comparison)	0.43	0.36	−0.28	1.14	1.21	0.228
time 2 (vs. 1)	−0.07	0.06	−0.19	0.05	−1.13	0.263
time 3 (vs. 1)	**−0.62**	**0.06**	**−0.74**	**−0.50**	**−10.32**	**<0.001**
time 3 (vs. 2)	**−0.55**	**0.06**	**−0.67**	**−0.44**	**−9.19**	**<0.001**
intervention * time 2 (vs. 1)	0.14	0.12	−0.10	0.38	1.15	0.253
intervention * time 3 (vs. 1)	0.19	0.12	−0.05	0.43	1.60	0.113
intervention * time 3 (vs. 2)	0.05	0.12	−0.19	0.29	0.45	0.652
**Oral Contraceptive**						
intervention (vs. comparison)	−0.20	0.16	−0.52	0.11	−1.28	0.205
time 2 (vs. 1)	**−0.07**	**0.02**	**−0.10**	**−0.03**	**−3.62**	**<0.001**
time 3 (vs. 1)	−0.02	0.02	−0.06	0.02	−1.11	0.269
time 3 (vs. 2)	**0.05**	**0.02**	**0.01**	**0.08**	**2.50**	**0.014**
intervention * time 2 (vs. 1)	0.02	0.04	−0.06	0.09	0.42	0.675
intervention * time 3 (vs. 1)	−0.01	0.04	−0.08	0.06	−0.25	0.803
intervention * time 3 (vs. 2)	−0.02	0.04	−0.10	0.05	−0.67	0.504

^†^Main effect and interaction estimates and p values are computed with contrast tests in the regression.

Interaction terms reference category is always control.

LB = Lower Bound, UB = Upper Bound for a 95% Confidence Interval.

**Table 5:** **Change in contraceptive sales by time and by pharmacy type (n = 84)**

**Condoms**					
**time 1-2**	**total**	**grocery comparison**	**independent intervention**	**grocery intervention**	
negative	42	20	11	11	
positive/zero	42	11	22	8	*Χ* ^2^ = 6.75, 2df, p = 0.034
**time 2-3**					
negative	62	30	15	17	
positive/zero	22	1	18	2	*Χ* ^2^ = 25.1, 2df, p < 0.001
**time 1-3**					
negative	58	29	12	17	
positive/zero	26	2	21	2	*Χ* ^2^ = 29.3, 2df, p < 0.001
**Oral Contraceptives**					
**time 1-2**	**total**	**grocery comparison**	**independent intervention**	**grocery intervention**	
negative	56	25	15	16	
positive	28	6	18	3	*Χ* ^2^ = 6.75, 2df, p = 0.034
**time 2-3**					
negative	35	10	17	8	
Positive	49	21	16	11	*Χ* ^2^ = 6.75, 2df, p = 0.034
**time 1-3**					
negative	48	21	16	11	
positive	36	10	17	8	*Χ* ^2^ = 6.75, 2df, p = 0.034

## Discussion

This study is among the first to use and evaluate community pharmacies as a communication channel for messages encouraging contraceptive use and promoting planned pregnancies. This study is important to the public health literature because of (1) a new communication channel, (2) positive findings and (3) future possible partnerships. The results showed that community pharmacies can be one mechanism for delivering messages related to using contraceptives. The social marketing materials placed in the pharmacies were conservative in appearance, passively delivered and modeled after previous materials. They were reviewed by pharmacists and the target group and approved by an advisory board. Using these materials for 7 quarters in 55 pharmacies from 12 Iowa counties, the intervention was associated with a decrease in the decline of condom sales, primarily in independent pharmacies. The results are based on a strong analytic approach with a comparison group and controlled for pharmacy type to examine the impact of the pharmacy social marketing campaign. We also controlled by age, gender, education and household size at the county level to control for factors that may differentiate the study groups and be associated with contraceptive use. A sensitivity analysis was also conducted in regards to data quality.

Stemming the reductions in condom sales in independent pharmacies is positive. It is likely that the messages in the pharmacies prompted individuals to purchase condoms. The messages may have been easier to notice in the independent pharmacies where the size of the pharmacy is smaller and the purchases are more focused on health products not groceries. It may be that some pharmacies became more focused on contraceptive products because of the study, and they began stocking condoms or offered more options. Admittedly, other sources of condoms such as convenience stores, mass merchandise stores and online purchases may be the likely cause of overall lower condoms sales in pharmacies over time. Stemming the decrease in condom sales in independent pharmacies is an important finding, as there are over 23,000 such outlets in the United States. The possibility of extrapolating this finding to chain pharmacies with over 50,000 outlets is important to consider in future studies.

The lack of impact on oral contraceptives was discouraging. It may be that the women in pharmacies who may have seen the messages were already using oral contraceptives. Alternatively, messages about how to obtain prescriptions for these products may be necessary. Targeting some of the materials, e.g., free product or brochure, to all women age 18–30 may have been more effective. However, targeting the materials would have required the pharmacy staff to alter their routine dispensing procedures, and this effect is difficult to achieve. Another important strategy may be to develop community pharmacy practices where women can gain access to hormonal contraceptives via pharmacist prescribing under a collaborative practice agreement. A limited number of pharmacies have experience using collaborative practice agreements to initiate hormonal contraceptives and such an approach may increase access to these products. This approach may be particularly relevant in rural communities such as those in Iowa included in this study [19,20].

It is important to note that during 2010 and early 2011, there was an active state-wide social marketing campaign called “Avoid the Stork” as part of the Iowa Initiative [24]. The pharmacy social marking campaign did not link directly to the “Avoid the Stork” campaign. In addition, there was also a state-wide investment in making long-acting reversible contraceptives available through family planning clinics. The overall results from the Iowa Initiative showed that unintended pregnancy rates decreased from 47.3% to 40.9% and that intra-uterine device and implant use increased among Iowa women from <1% to 7.45 and 6.0%, respectively. The use of the comparison pharmacy sales data allowed us to separate the impact of other campaigns on any effect we saw in the intervention pharmacies [21].

This study serves as a model to involve community pharmacies in public health campaigns, including those focused on reducing unintended pregnancy. A limitation of this study is that national, chain pharmacies did not participate because either the messages were perceived to be too sensitive or the companies did not want to share their sales data. Their lack of participation was disappointing because all of those pharmacy organizations sold contraceptives, and messages about their use in preventing unintended pregnancy are important to public health. Their participation in future initiatives should be encouraged because the inclusion of the top two pharmacy chains impacts just over 30% of the prescription market share. A rationale for participation in such initiatives that may possibly garner corporate support is to link public heath campaigns to corporate social responsibility.

Future projects focused on reducing unintended pregnancy or other public health issues should consider coordinating messages with community-based initiatives of public health departments or health systems, and this coordination might facilitate their effectiveness. In addition, further testing of the messages and marketing pieces with the target population may be needed. As well, involving pharmacy technician and pharmacists more fully into any future intervention would likely be necessary if the desire was to create more extensive behavior change among pharmacy clients.

This study had limitations. A comparison group was used but neither the counties nor pharmacies were randomized. Measurement error may have occurred in the sales data by mistakes in reporting. One sensitivity analysis accounted for some apparent missing data, and the analysis showed no effect of excluding these pharmacies on the results. As stated, only independent and grocery pharmacies were included in this study, thereby limiting the generalizability of the findings. Finally, there was no explicit focus on emergency contraception in this study, although access to these products and how to use them are important public health roles for pharmacists.

## Conclusions

Contraceptive sales data were used to quantify the impact of a passive social marketing intervention in community pharmacies before and during the intervention. While condom sales were decreasing in both study groups, the intervention appeared to stem the reduction in sales in the intervention pharmacies, particularly independent pharmacies. Oral contraceptive sales were not affected.

## Abbreviation

s.d: Standard deviation
